# Modern contraceptive use among reproductive-aged women in Ghana: prevalence, predictors, and policy implications

**DOI:** 10.1186/s12905-018-0649-2

**Published:** 2018-09-25

**Authors:** Paul Beson, Richard Appiah, Augustine Adomah-Afari

**Affiliations:** 1 MPH, BSN, Lekma Hospital, Greater Accra Region, Accra, Ghana; 2 Africa Unit for Transdisciplinary Health Research, North-West University, Potchefstroom, South Africa; 3 Department of Occupational Therapy, College of Health Sciences, University of Ghana, Accra, Ghana; 4 Department of Health Policy, Planning and Management, School of Public Health, University of Ghana, Accra, Ghana

**Keywords:** Modern contraceptives, Prevalence, Predictors, Reproductive-aged women, Ghana

## Abstract

**Background:**

Modern contraceptive use remains an important public health intervention and a cost-effective strategy to reduce maternal mortality, avert unintended pregnancies and to control population explosion, especially in developing countries. Despite these benefits, there are reports of low usage among reproductive-aged women in most developing countries. This study examined the prevalence and predictors of use of modern contraceptive among reproductive-aged women in an urban center with a high density population in Ghana.

**Methods:**

A cross-sectional, interviewer-administered survey was conducted with 217 randomly selected reproductive-aged women. Data was analyzed with STATA. Logistic regression was performed to identify factors influencing modern contraceptive use.

**Results:**

Although we found high levels of knowledge and awareness (98%; *n* = 213) of modern contraception use, only 21% of participants were using modern contraceptives. Marital status, partner consent and support, and religious beliefs strongly predicted usage.

**Conclusion:**

Usage of modern contraceptives among reproductive-aged women in the Ledzokuku Krowor Municipality is lower than the national target. A multilevel family planning intervention program that primarily focuses on promoting inclusive participation of husbands, targets the unmarried and non-literates reproductive-aged women, and dispels misconceptions, misinformation and religious myths about modern contraceptives has been discussed.

## Background

Unintended pregnancy is a well-established public health concern with high proclivity among sexually active women [1]. Globally, an estimated 40% of women report with unintended pregnancies [2]**.** Modern contraceptives remain an effective method of preventing unintended pregnancies [3, 4]. However, in spite of the wide range of effective modern contraceptive options available to women with its benefits, global statistics indicate low usage with increasing unintended pregnancies both in developed and developing countries [4]. In Africa, particularly in sub-Saharan Africa (SSA), research evidence consistently reports low prevalence of use of modern contraception, which translates to high incidence of unintended pregnancies, unsafe abortions, and maternal deaths [4–6]. Poor contraceptive use has mortality and clinical implications. Data from developing countries suggest that a woman dies every eight minutes from unsafe abortion arising from unplanned pregnancies [1]. Previous research in Ghana, similar to other SSA countries, reports prevalence rates lower than the national target. In a recent effort, researchers found a prevalence of use at 17% in public and private health facilities in a peri-urban community in Ghana, which is lower than the national target of 23.3% [7].

There is evidence to suggest that induced abortion and its related complications are the most common outcomes of non-use of modern contraceptives. For instance, induced abortions account for about 12% of maternal deaths in Ghana, ranking third after hemorrhage (22%) and unclassified causes (14%) [8]. Maternal deaths are estimated to be 1.8 times higher in women without modern contraceptive use compared to users [8]. Prior research shows that most women in SSA, and Africa more generally, are confronted with several contextual and administrative challenges in their efforts to access and use modern contraceptives [4]. For instance, fear of side effects from contraceptives due to inadequate knowledge was found to be a leading cause of non-use of modern contraceptives in rural Ghana [8]. However, within the Talensi district of the Upper East Region of Ghana, researchers found partner’s disapprovals as the major reason for non-use of modern contraceptives by women [4]. These differences suggest that the decision to use modern contraceptives is influenced by several factors, which are often contextual.

The benefits of modern contraception have long been established. It is estimated that up to 35% of maternal deaths and 13% of child mortalities could be averted whilst 25% of under-five mortalities could be prevented if birth intervals were at least three years and by the use of various contraceptive methods in planning their families [5]. A wealth of research also demonstrate that employing various methods of modern contraceptives in planning families promote gender equality as well as educational and economic empowerment for women [4]. Utilization of modern contraceptives among reproductive-aged women could have important policy and health care cost implications for poverty reduction and socio-economic development in developing countries. In an empirical study [8], researchers found that for every dollar spent on modern contraceptives in family planning, at least US$4 that could have been spent treating complications from unintended pregnancies is saved, whilst governments also save up to US$31 in health care cost [8]. In furtherance to this, consumers of modern contraceptives take control over their reproductive capacity and free themselves from the anxiety of involuntarily becoming pregnant, and therefore enable them to have a fuller enjoyment of their sexuality [9].

Previous researches have explored the factors influencing modern contraceptive use in Ghana [3–5, 7, 10]. However, these studies were predominantly conducted in rural settings and included pilot projects in the country’s northern regions. As far as could be established, presently, there is a dearth of research data from densely populated urban communities of Ghana [11, 12]. Findings from this study will provide data on factors that predicts modern contraception use and will guide researchers and clinicians working to develop contextually relevant intervention programs to advance the use of modern contraception, particularly in densely populated urban settlements. The findings could also inform policy formulation and implementation of effective family planning programs that support women and their partners to plan their pregnancies and families and to have fewer and healthier children thereby reducing socio-economic burden.

## Methods

### Study setting

The Greater Accra Region is both **the smallest and the most populated of Ghana’s 10 administrative regions,** with an estimated population of 4,010,054, which accounts for 16.3% of Ghana’s total population as at 2010 [13]. This study was conducted at the Ledzokuku Krowor Municipality (LEKMA), a highly populated 50 km^2^ land area bounded by the Gulf of Guinea in the south and wedged between the Accra Metropolitan Assembly on the west and Tema Metropolitan Assembly on the east [12]. The municipality has 82 communities with an estimated population of about 500,000 representing 0.92% of the total national population of 29,658,823 and 5.7% of the Greater Accra’s total population of about 4,010,054 respectively. Females account for about 51% of the population of LEKMA, with about 27% in their reproductive age [12]. Residents are predominantly traders with limited employment opportunities. The Ledzokuku Krowor Municipal hospital, which is the biggest hospital in the municipality, provides both in- and out-patient services, including reproductive health services to residence.

### Study design, participants and sampling

A cross-sectional survey was conducted from June to July of 2016. Using Cochran [14] formulae with a level of precision of 0.05, and the national modern contraceptive prevalence of 17% [13] sample size of 217 was determined for the study. The study sample was drawn from a subsample of reproductive-aged women who were sexually active, resident at LEKMA and accessing health services at the LEKMA hospital. Participants were recruited by simple random sampling through the ballot method. All reproductive-aged women who accessed services at the time of data collection were introduced to the study and invited to participate. Consented individuals were asked to pick from a box containing pieces of papers with numbers. These pieces of papers were collected back from them and their respective numbers noted against their names. At the close of each working day, the papers were put into a container and thoroughly mixed and then picked out one by one at random until the calculated sample size apportioned for the particular day was drawn, and repeated each day until the total sample size was reached. Individuals whose numbers were drawn were contacted and scheduled for interview. To ensure that participants’ present medical conditions do not influence their responses, only individuals who presented with general medical conditions which were unrelated to use of modern contraception were selected.

### Study instrument and data collection

A structured interviewer-administered questionnaire was designed in English language and administered by trained data collectors who equally understand the three dominant Ghanaian languages (Akan, Ewe and Ga) in the study area to enable them explain the questionnaire to illiterates and semi-literate participants. The questionnaire was designed by the researchers after reviewing literature and in consultation with two independent expert analysts from the School of Public Health, University of Ghana. Experts’ ratings were statistically analyzed to determine content validity. A pre-test of the study instrument was conducted with 40 reproductive-aged women recruited from another district hospital to correct items that were found to be ambiguous. The instrument collated data on the socio-demographic characteristics of study participants, knowledge of and awareness of modern contraceptives and use of modern contraceptives. Data quality was ensured through onsite supervision, spot-checks, and back-checks of collated data.

### Data analysis

Data was coded, entered into Microsoft Excel, exported to STATA (version 13.0) and analyzed. Participants’ background data was analyzed using descriptive statistics. Chi-square test and logistic regression models (bivariate and multivariate) were employed to assess level of significance and the association between the dependent and exposure variables.

## Results

### Socio-demographic characteristics of study participants

Most participants (68%) were married, aged between 20 and 29 years (55%), and had obtained a Junior High School level of education (31%). Majority of the participants had had between 1 and 3 conceptions (68%), had no specific income levels (67%), and were Christians (87%). Detailed socio-demographic data of participants is presented in Table 1 below.

**Table 1:** Socio-demographic characteristics of participants (*N* = 217)

Variable	Frequency (N)	Percentage (%)
Age		
15–19	23	11
20–29	120	55
30–39	69	32
40–49	5	2
Marital Status		
Single	63	29
Married	147	68
Divorced	4	2
Widowed	3	1
Educational level		
Primary	28	13
JHS	66	31
SHS	53	24
Tertiary	53	24
None	17	8
Conception		
1–3	147	68
4–6	42	19
7–9	0	0
None	28	13
Employment		
Trader	65	30
Civil Servant	39	18
Others	75	35
None	38	17
Monthly Income		
50–300	22	10
301–600	24	11
601–1000	14	7
1001 above	11	5
Can’t tell	146	67
Religion		
Christianity	189	87
Islamic	14	7
African Traditional Religion	5	2
Others	9	4
Total	217	100

### Prevalence of current use of modern contraception and background characteristics of women

The overall prevalence of use of current modern contraceptive was 21%. Table 2 shows the results of the proportions of prevalence of modern contraceptive use and background characteristics of participants. Among married women, there was 29% prevalence of current use of modern contraceptive compared to only 5% among single women. Women who received partner support reported high prevalence (34%) of use of modern contraceptives.

**Table 2:** Prevalence of current contraceptive use and background characteristics of women (N = 217)

Variable	Frequency N(%)	Current use of modern contraceptives	
		Yes- N(%)	No-N(%)
Age			
15–19	23 (11)	0	23 (100)
20–29	120 (55)	28 (23)	92 (77)
30–39	69 (32)	17 (25)	52 (75)
40–49	5 (2)	1 (20)	4 (80)
Marital status			
Single	63 (29)	3 (5)	60 (95)
Married	147 (68)	43 (29)	104 (71)
Divorced	4 (2)	0	4 (100)
Widowed	3 (1)	0	3 (100)
Education			
Primary	28 (13)	5 (18)	23 (82)
JHS	66 (30)	17 (26)	49 (74)
SHS	53 (24)	11 (21)	42 (79)
Tertiary	53 (24)	11 (21)	42 (79)
None	17 (9)	1 (6)	16 (94)
Conception			
1–3	147 (68)	36 (24)	111 (75)
4–6	42 (19)	8 (19)	24 (57)
7–9	0	0	0
None	28 (13)	2 (7)	26 (93)
Employment			
Trader	65 (30)	15 (23)	50 (77)
Civil servant	39 (18)	10 (26)	29 (74)
None	75 (35)	8 (11)	67 (89)
Others	38 (17)	13 (34)	25 (66)
Income			
50–300	22 (10)	5 (23)	17 (77)
301–600	24 (11)	6 (25)	18 (75)
601–1000	14 (7)	6 (43)	8 (57)
1001above	11 (5)	2 (18)	9 (82)
Can’t tell	146 (67)	27 (18)	119 (82)
Religion			
Christianity	189 (87)	41 (22)	148 (78)
Islamic	14 (7)	4 (29)	10 (71)
ATR	5 (2)	0	5 (100)
Others	9 (4)	1(11)	8 (89)
Partner support			
Yes	71 (33)	24 (34)	47 (66)
No	114 (53)	15 (13)	99 (87)
Not discussed	32 (14)	7 (22)	25 (78)

Yes- N(%) = number of participants who responded “Yes” to current use of modern contraceptives

No-N(%) = number of participants who responded “No” to current use of modern contraceptives

### Knowledge of and source of information on modern contraceptives

We found a high level of knowledge (the ability to mention at least one type of modern contraceptive method) and awareness of modern contraception methods. The result showed that majority of participants (98%; *n* = 213) had adequate knowledge (the ability to mention at least one type of modern contraceptive method) of modern contraceptives and knew at least a method of modern contraception. Majority of participants (64.5%; *n* = 140) identified the injectable as a modern method of contraceptive. This was followed by the pills (61.8%; *n* = 134). The least known method was the diaphragm (2.8%; *n* = 9). The television was the major source (56%; *n* = 122) of information on modern contraceptives. This was followed by the hospital (38%; *n* = 83). The newspaper/magazines constituted the least (0.5%; n = 1) source of information on modern contraceptive. Details of levels of knowledge and awareness and sources of information on modern contraceptives are presented in Table 3.

**Table 3:** Knowledge and awareness of modern contraceptive (*N* = 127)

Variable	Frequency	Percentage (%)
Methods		
Male condom	131	60.4
Female condom	84	38.7
Injectable	140	64.5
Pills	134	61.8
Implants	75	34.6
IUD	34	15.7
Foam/Jelly	9	4.1
Diaphragm	6	2.8
Male sterilization	19	8.8
Female sterilization	17	7.8
Source of knowledge		
Television	122	56.2
Radio	56	25.8
Hospital	83	38.2
Friends	31	14.3
Posters/Banners	5	2.3
Newspapers/Magazines	1	0.5
Community social clubs	8	3.7
School	11	5.1

### Predictors of modern contraceptive use

The results reveal that participants with positive attitude towards modern contraceptives were significantly more likely to use modern contraceptives, compared to those with negative attitude. This was found for both bivariate (OR = 4.2, 095% CI: 1.8–9.8) and multivariate (OR = 3.9, 095% CI: 1.7–9.4) analysis. Furthermore, use of modern contraceptives was also predicted by one’s religious beliefs. Participants who factored in religious beliefs when deciding about modern contraceptives use, were less likely to use modern contraceptive (OR = 0.4, 095% CI: 0.2–0.9), compared to those who do not consider their religious beliefs when deciding on modern contraceptive use. Table 4 presents the results of the logistic regression.

**Table 4:** Logistic regression of factors influencing modern contraceptive use among reproductive age women (n = 217)

Variable	Modern contraceptive use	Bivariate O.R.(95% CI)	Multivariate O.R.(95% CI)
Knowledge and awareness	Yes	0.8 (0.1–7.9)	0.7 (0.1–8.8)
	No	1.00	
Attitude	Yes	4.2 (1.8–9.8)**	3.9 (1.7–9.4)**
	No	1.00	
Knowledge of source of availability	Yes	1.9 (0.2–16.0)	2.2 (0.3–19.0)
	No	1.00	
Religious influence	Yes	0.4 (0.2–0.9)*	0.5 (0.2–0.9)*
	No	1.00	

Notes: *Denote p-value < 0.05 and **p-value < 0.01

## Discussion

We sought to determine the prevalence of use of modern contraceptive, knowledge of modern contraceptives, and predictors of modern contraceptive use among reproductive-aged women in a densely populated municipality of Ghana. We found the overall prevalence of use at 21%, similar to the 22% reported by the Ghana Demographics and Health Survey [12], but below the Ghana Health Service’s national family planning target rate of 23.3% [4]. A previous study conducted in Iran reported usage of modern contraceptive at 55% [15]. This higher rate, compared to the 21% found in this study, could be attributed to the availability of a well formulated and coordinated programs aimed at eliminating both cultural and economic barriers to family planning in Iran. For instance, in Iran, population education is part of the curriculum at all educational levels, including university and colleges, where students are required to take a two-credit course on population [15]. Although family planning education is available in Ghana, this is limited to healthcare facilities and on national media [5]. Educational programs with relevant practical demonstrations of use of modern contraceptives in various Ghanaian dialects ought to be developed, evaluated, and scaled-up to all schools, marketplaces, and communities to augment existing reproductive health programs.

The results also showed a high level (98%) of knowledge and awareness of modern contraceptives among the reproductive-aged women enrolled in the study. This finding corroborates with the 99% level of knowledge and awareness of modern contraceptive reported in Ethiopia [16] and India [9]. This widespread knowledge of modern contraceptives could be partly due to the various behaviour change communication or social marketing strategies in the form of visual and audio advertisements and educational interventions put in place to promote contraceptive use in the Ghanaian society [9].

Our findings also portrayed a non-lineal relationship between knowledge and awareness and current use of modern contraceptive, implying that knowledge and awareness per se do not drive modern contraceptive use. Several studies have consistently reported negative correlation between widespread knowledge and awareness of modern contraceptives and use of same [12, 16]. Fear of side effects, myths and misconceptions about modern contraceptive as well as service provider factors have been associated with the low level of use, despite the high knowledge and awareness levels [11].

Contrary to previous research in Nigeria which reported friends as the major source of contraceptive knowledge or information among a group of market women of reproductive-aged at 33.8% [17], the results indicated the television was the main source of contraceptive knowledge (56.2%) in this study. This difference may be due to the fact that the participants in the Nigeria study were purely a cohort of market women of reproductive age. Thus, by their commonality of trade, this may foster information sharing, including information on modern contraceptives. Although sharing of information regarding contraceptives by word of mouth among friends is recognized as equally important particularly in settings of less education [17], it is argued that such information is more likely to be shrouded with misinformation, distortion, falsehoods, and misconception, and may be self-centered [18]. Given that television is regarded as more reliable and educative compared to information from friends [17], this finding is more instructive, comparatively. In another study to explore the source of contraceptive knowledge or information among reproductive-aged women in the Nkwanta district of Ghana, Eliason and colleagues identified health workers as the main source [5]. Given that communities of the Nkwanta district are predominantly rural with poor television and radio receptions, the community-based health planning and services (CHPS) system in the communities provide basic health services and health information to residents. In contrast to this setting, the present study was conducted within an urban, densely populated municipality of Accra where most participants are rather individualistic, but with access to television and other media [17].

The results indicated the injectable as the most widely known method of modern contraceptive among respondents (64.5%). This was followed by the pills (61.8%), with the diaphragm (2.8%) as the least known method. This finding parallels a previous case control study in Ghana, which found the injectable as the most known method of modern contraception amongst both cases (93.1%) and controls (82.6%) [5]. Previous research in Ghana found that use of contraceptives is associated with promiscuity [19]. As a result, users, as indicated in previous and in this study, seeks information on and prefers to use the injectable, which is deemed to be a more covetous method. Previous studies that explored the availability of modern contraceptives in public and private health facilities in Ghana [5, 7] as well as studies in Ethiopia [20] also reported the injectable as the most widely known method of modern contraceptive. This finding, however, contradicts earlier survey in Ghana that reported the male condom as the most known method of contraceptive [12], and in India where researchers found female sterilization as the most widely known method at 97.7% [9].

Our results found no significant association between level of formal education and current use of modern contraceptive. This may reflect the general educational curriculum of Ghana, which is often criticized as lacking reproductive health education components, especially at the primary and basic educational levels [21]. This finding contradicts the wealth of evidence from other parts of Africa that reported formal education as a strong predictor of modern contraceptive use [10, 22, 23]. Since formal education of women is considered an important strategy towards promoting contraceptive use, our finding has important policy implications. Health policy makers and government and non-governmental agencies need to evaluate, re-strategize and promote the role of education in Ghana’s effort to achieve the 50% target of modern contraceptive use by 2035.

Similar to previous findings from Nigeria [17] and Uganda [24], the results showed that marital status of women strongly predicted use of modern contraceptives (*p* < 0.001). It was previously established that most women who used modern contraception (about 70%) did so for purposes of spacing births, and women who did not have children were least likely to use contraceptives [25]. However, other findings suggest that unmarried individuals use modern contraceptives to prevent pregnancies [26].

Furthermore, partner support was strongly associated with current use of modern contraceptives. This reflects the generally dominant and influential role of men in decision making in the predominantly traditional patriarchal societies of Ghana. This state is reinforced by patriarchal values systems that marginalizes and de-emphasizes female empowerment and independence in decision making, including reproductive health decisions [16, 24, 27, 28]. This confirms previous empirical study in Ghana that, partners’ consent and approval to the use of modern contraceptive methods was crucial to the success of family planning intervention efforts [5]. Study outcomes from peri-urban settings of Ethiopia, Ghana, and Uganda also revealed that since women are expected to be sexually passive to safeguard their dignity and honor in the African setting, they surrender decisions on contraceptive use to the discretion and approval of their partners [24, 27, 29]. Consistent with findings of a previous study in Ghana [10], employment status of study participants also significantly predicted use of modern contraceptives (*p* < 0.05).

In addition, majority (63%) of participants portrayed a positive attitude towards modern contraceptive use. Our study further revealed that women who had positive attitude towards modern contraceptives were 4.2 times more likely to use modern contraceptives compared to those who do not (OR = 4.2, CI: 1.8–9.8) in bivariate analysis. This was still significant after multivariate analysis (OR = 3.9, CI: 1.7–9.4), suggesting a strong relationship between attitude and use of modern contraceptives.

Religiosity also strongly predicted use of modern contraception. Participants who consider their religious beliefs in making decisions about modern contraceptives use, had less odds of 0.4 times of using modern contraceptives compared to those who would not consider their religious beliefs in their contraceptive decisions in both bivariate and multivariate analysis (OR = 0.4, CI: 0.2–0.9) (OR = 0.5, CI: 0.2–0.9) respectively. This contradicts findings from previous empirical study in Uganda that reported religious influence as a significant predictor of use of a modern contraception [30]. This divergence in findings is striking in view of the fact that one would have thought that religious beliefs would rather influence decision not to use modern contraceptives, as chastity is required of staunch religious practitioner. However, the fear of getting pregnant or contracting sexually-transmitted infections and being reprimanded by one’s religious affiliation and doctrines rather motivates use. It thus suggest that people may prioritize contraceptive use over religious beliefs as long as they understand the benefits such as preventing unwanted pregnancies and sexually transmitted diseases [31].

### Study limitation

This study only recruited women who were accessing healthcare services at LEKMA. Furthermore, the instruments used for data collection were designed by the researchers. The instruments were however critically reviewed by experts and pilot-tested before they were administered.

## Conclusion

This study sought to determine the prevalence of use, level of knowledge, and predictors of modern contraceptive use among reproductive-aged women in the Ledzokuku Krowor Municipality, a densely populated residential part of the Greater Accra Region. The findings revealed that although there was a high level of knowledge and awareness of modern contraceptives, only a small number of women actually use them. A number of factors, including partners’ approval, marital status, and employment status were associated with use of modern contraceptives while only religious beliefs and attitude significantly predicted use of modern contraceptives. Given these outcomes, we propose that the Ministry of Health, the Ghana Health Service, the Ministry of Education, and agencies involved with reproductive health programs should focus on developing behavior change communication strategies in the various Ghanaian languages and dialects aimed at fostering positive attitude towards modern contraceptives among women instead of the present preoccupation with creation of knowledge and awareness about modern contraceptives through various educational programs. New efforts should also necessarily target dispelling the misinformation, misconceptions, and wrong religious beliefs and myths surrounding the use of modern contraception. In particular, the use of influential and religious individuals as ambassadors of modern contraceptive use will help to reshape people’s religious perspective on modern contraceptive use and foster positive attitude toward usage. The Ministry of Education and Ghana Education Service should introduce and intensify reproductive health information in the curricula at basic and secondary school levels to provide adequate education and help empower children against non-liberal religious beliefs on modern contraceptive use which are introduced to children as they develop sexually. This will help create a critical mass of citizens with positive attitude relative to modern contraceptive use to help drive Ghana’s efforts towards achieving the 50% target of modern contraceptive use by 2035.

## Abbreviations

CHPS: Community based Health Planning and Services
CI: Confidence Interval
LEKMA: Ledzokuku Krowor Municipal Assembly
OR: Odd Ratio
SSA: sub-Saharan Africa
US: United State
US$: United State Dollars
